# Extracorporeal membrane oxygenation bridging for chemotherapy in obstructing mediastinal mass after cardiopulmonary arrest

**DOI:** 10.1186/s13019-024-02918-1

**Published:** 2024-06-26

**Authors:** Daniel Wilkinson, Enoch Yeung, Sanjay Samy, Chikashi Nakai

**Affiliations:** Department of Cardiothoracic Surgery, Albany Medical Center, Albany, NY USA

**Keywords:** Extracorporeal membrane oxygenation, Mediastinal mass, Chemotherapy

## Abstract

**Background:**

In a sedated patient, airway compression by a large mediastinal mass can cause acute fatal cardiopulmonary arrest. Extracorporeal membrane oxygenation (ECMO) has been investigated to protect the airway and provided cardiopulmonary stability. The use of ECMO in the management of mediastinal masses was reported, however, the management complicated by cardiopulmonary arrest is poorly documented.

**Case presentation:**

32-year-old female presented with acute onset of left arm swelling and subacute onset of dry cough. Further investigation showed a deep venous thrombosis in left upper extremity as well as a large mediastinal mass. She underwent mediastinoscopy with biopsy of the mass which was complicated by cardiopulmonary arrest secondary to airway obstruction by the mediastinal mass. Venoarterial ECMO was initiated, while concurrently treating with a chemotherapy. The mediastinal mass responded to the chemotherapy and reduced in size during 2 days of ECMO support. She was extubated successfully and decannulated after 2 days of ECMO and discharged later.

**Conclusions:**

Extracorporeal membrane oxygenation can serve as a viable strategy to facilitate cardiopulmonary support while concurrently treating the tumor with chemotherapy, ultimately allowing for the recovery of cardiopulmonary function, and achieving satisfactory outcomes.

## Background

An anterior mediastinal mass significantly increases the risk of airway compromise and circulatory arrest [1]. In a sedated patient, airway compression by a large mediastinal mass can cause acute fatal cardiopulmonary arrest. Extracorporeal membrane oxygenation (ECMO) has been investigated to protect the airway and provided cardiopulmonary stability. However, the use of ECMO in the management of mediastinal masses is poorly documented.

## Case presentation

**Fig. 1:**
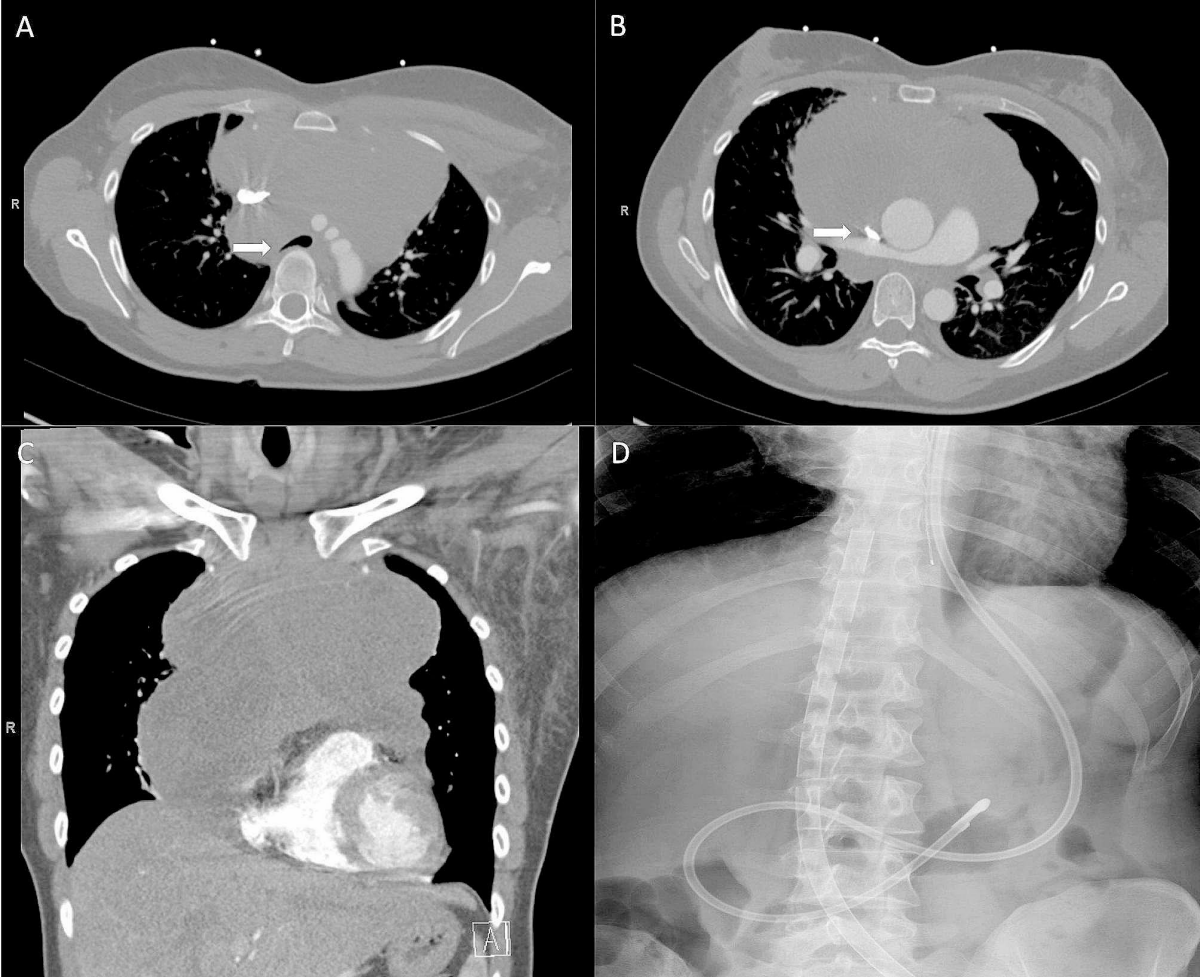
Pretreatment chest computed tomography (**A**) a mediastinal mass compressing the distal trachea against the vertebral column (white arrow). (**B**) Stenosis of the superior vena cava compressed by a mediastinal mass (white arrow). (**C**) a large heterogeneous 12 × 8 × 14 cm soft tissue mass within the anterior mediastinum (**D**) Xray abdomen after VA ECMO cannulation

**Fig. 2:**
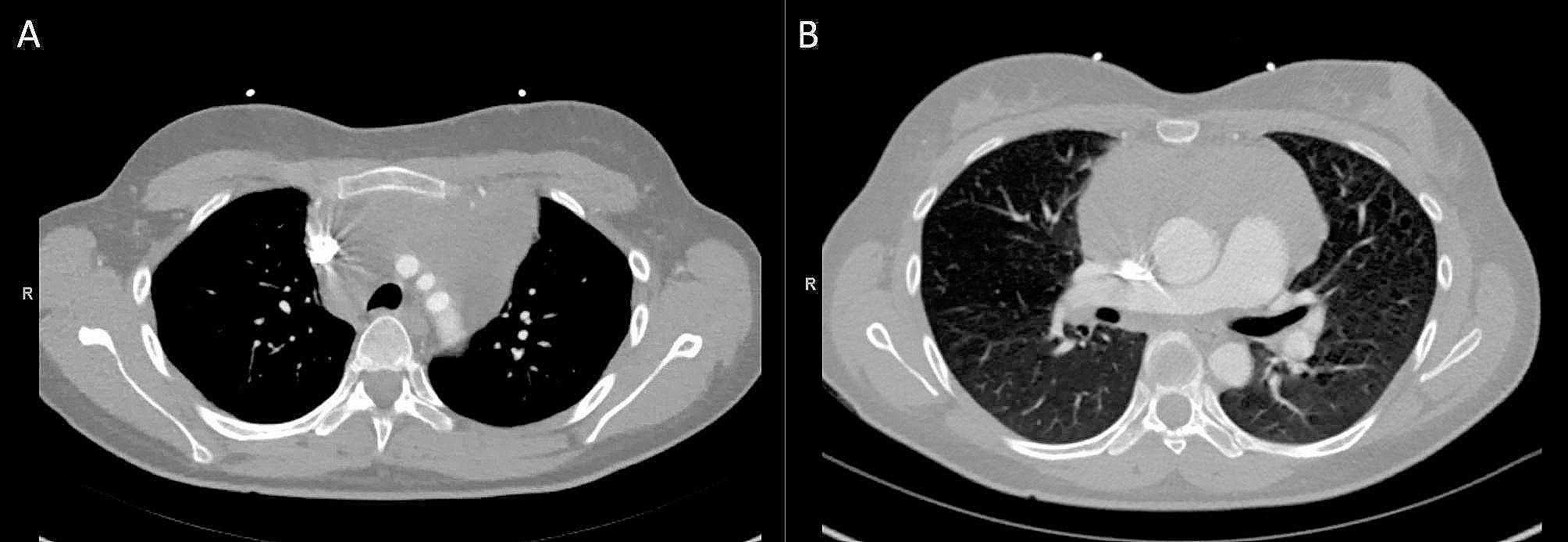
Posttreatment chest computed tomography (**A, B**) A heterogeneous 11 × 6 × 10 cm soft tissue mass within the anterior mediastinum. Decreased size and mass effect compared to pretreatment chest computed tomography

This previously healthy 32-year-old female presented to the emergency department with one day of left upper extremity (LUE) swelling, two-month history of dry cough, and one-year history of progressive exertional dyspnea. Further investigation revealed an acute LUE deep venous thrombosis (DVT) as well as a large mediastinal mass. Computer tomography revealed a large heterogeneous 12 × 8 × 14 cm soft tissue mass within the anterior mediastinum with local mass-effect on the heart resulting in compression of the right main pulmonary artery and distal trachea against the vertebral column (Fig. 1). There was an acute LUE DVT and stenosis of superior vena cava (SVC) and right distal subclavian vein (Fig. 1). Heparin drip was initiated, and she was transferred to our facility for a mediastinoscopy with biopsy of the mass. Induction of general anesthesia and intubation were performed without any issues. She tolerated ventilation during her uncomplicated mediastinoscopy. After the surgical team and anesthesiologists discussed the timing of extubation, the decision was made to proceed with extubation after the procedure. However, several minutes after extubation, she developed profound hypoxia refractory to bag mask ventilation and went into cardiopulmonary arrest (CPA). One minute of cardiopulmonary resuscitation (CPR) was required prior to achieving return of spontaneous circulation (ROSC). Reintubation was performed during this time and right femoral arterial and left femoral venous access was obtained. The decision was made to hold off on placing ECMO cannulas at this point since she was easily able to be ventilated with normal oxygenation. However, as her sedation wore off, the second CPA secondary to profound hypoxia happened while she was still intubated. One minute of CPR was performed and she was re-sedated and paralyzed with subsequent ROSC. Venoarterial (VA) ECMO was chosen to support her heart function damaged by two CPA events and due to the fact that the superior vena cava (SVC) was compressed by the mass and not suitable for internal jugular venous return in VV ECMO. An arterial cannula was placed in the right femoral artery with a distal perfusion cannula and a venous cannula was placed in the left femoral vein (Fig. 1). VA ECMO was initiated. A preliminary diagnosis of lymphoma was made, and she was initiated on EPOCH chemotherapy regimen and steroids on the evening of the cannulation of ECMO. Her hemodynamics and respiratory status were clinically improved after the initiation of the chemotherapy. She was successfully extubated and decannulated on post ECMO day 2. The final pathology demonstrated B cell lymphoma. Repeated CT on post ECMO day 11 showed significant reduction in size of the mediastinal mass (Fig. 2). She was discharged home on post ECMO day 12 on apixaban for her DVT with plans to continue her EPOCH regimen.

## Discussion and conclusions

We present the use of ECMO as a bridge to urgent chemotherapy. Emergent ECMO has been documented in several case reports involving patients with mediastinal masses [2]. ECMO was indicated in the patient because the patient sustained two circulatory arrests caused by hypoxia: the first after extubation and the second after re-intubation due to compression of her trachea distal to the endotracheal tube. We could not therefore extubate her without first shrinking her mediastinal mass. Frey TK et al. reported the use of ECMO in a patient receiving chemotherapy [3]. Two ECMO options are available: venoarterial (VA) and venovenous (VV). In our case, considering the presence of cardiac arrest and compressed SVC, VA ECMO was deemed beneficial for both cardiac and respiratory support. However, in the absence of cardiac issues, VV ECMO with endoscopic stenting for airway protection could be an alternative option [4, 5]. In our case, the mass compressed the airway from the distal trachea to right and left main bronchi. The compression area was too distal and long to place on stent.

Currently, there are no established guidelines for the rescue use of ECMO in cases involving mediastinal masses. Nevertheless, some authors have suggested implementing a contingency plan to mitigate potential complications. They recommended placing 5 Fr sheaths in the femoral vein and artery before intubation in high-risk patients to enable swift cannulation in the event of unstable hemodynamics [6]. We concur with this approach, as using a guide wire during placement may facilitate rapid cannulation. Another factor to consider in this case is the possibility of tumor lysis syndrome (TLS), which often occurs following the initiation of cytotoxic therapy in patients with clinically aggressive and highly aggressive tumors such as lymphoma. TLS is characterized by the massive lysis of tumor cells, releasing large amounts of potassium, phosphate, and nucleic acids into the systemic circulation [7]. In our case, the patient responded well to chemotherapy and there was no siginificant issue related to TLS while on VA ECMO support. In some cases, ECMO might be crucial in managing specific TLS-related consequences, including electrolyte abnormalities [8]. 

This case is an example of a patient experiencing significant respiratory compromise secondary to mechanical compression from a mediastinal mass, resulting in CPA. We have demonstrated that VA ECMO can serve as a viable strategy to facilitate cardiopulmonary support while concurrently treating the tumor with chemotherapy, ultimately allowing for the recovery of cardiopulmonary function, and achieving satisfactory outcomes.

## Data Availability

No datasets were generated or analysed during the current study.

## Abbreviations

ECMO: Extracorporeal membrane oxygenation
LUE: Left upper extremity
DVT: Deep venous thrombosis
SVC: Superior vena cava
CPA: Cardiopulmonary arrest
CPR: Cardiopulmonary resuscitation
ROSC: Return of spontaneous circulation
VA: Venoarterial
VV: Venovenous
